# A biotechnologist's dream: ‘doubly green’ processes

**DOI:** 10.1111/1751-7915.12762

**Published:** 2017-07-11

**Authors:** Ana Segura

**Affiliations:** ^1^ Estación Experimental del Zaidín CSIC Profesor Albareda 1 18008 Granada Spain

## Abstract

Biosurfactants have not fulfilled the expectations generated when they were initially discovered. Heavy vacuum gas oil (HVGO) is conventionally treated with costly thermo‐chemical processes and cause environmental pollution. What if we could bio‐upgrade this HVGO at the same time that we produce a fine chemical?

Surfactants are used in a wide variety of industries; such as the production of paints, plastics, paper and agricultural products, the food and fine chemical industry as well as in the production of personal care products. The surfactants market was valued at 20 billion dollars in 2006 and is expected to reach 40 billion dollars by 2020 (Janshekar *et al*., 2007; http://www.marketsandmarkets.com/PressReleases/surfactants.asp, 2016). Despite the good properties of biological surfactants, currently most of the commercial surfactants are synthetic. Among the advantages of the use of biological surfactants *versus* chemically synthesized ones are that they are non‐toxic and they are biodegradable, therefore, environmentally friendly. Because of their natural origin, in many cases, they show biocompatibility making them particularly relevant in the cosmetic, pharmaceutical and food industries (Kosaric, 1992). However, the high production costs of the biological congeners are limiting their production on a large scale and therefore their commercial use (Banat *et al*., 2014). High yields and high biosurfactant concentrations in bioreactors are essential for their recovery; one of the main problems encountered for the industrial production of biosurfactants is posed by the downstream processing of bioreactor diluted broths as pure compounds are required for many applications. The optimization of the factors that affect the growth of biosurfactant‐producing microorganisms is therefore key for their economically viable production. Among the different studies carried out to define the best growth conditions, including the definition of the best ratio between carbon, nitrogen, phosphorus and iron needed to obtain high biosurfactant production yields, the utilization of waste substrates to balance the overall production costs has been envisaged as a promising option to boost biosurfactant production. However, most of these studies have been focused towards the use of industrial glycerol, animal fat or agricultural wastes (Makkar *et al*., 2011), and the production from petroleum residues has not so far been thoroughly investigated.

Moreover, petroleum refinery industries have started to upgrade heavy oils and residues to cope with the increasing demand of fuels, energy and petrochemical products (Sahu *et al*., 2015). In petroleum refining industries, the atmospheric distillation unit is used to extract naphta, kerosene, diesel and gas oil. Residues from this first treatment are transferred to a vacuum distillation unit, where light and heavy vacuum oil are extracted (LVGO and HVGO) and a vacuum residue (VR) is generated (Fig. 1). Whilst LVGO, depending on its properties, can be blended with other products, HVGO is normally used as feed for fluid catalytic cracking units to obtain an upgraded product that could be distillated to obtain fuel. HVGO upgrading processes require high temperature and pressure and huge amounts of catalysts, therefore this is an expensive, energy intensive and contaminating process (Sahu *et al*., 2015). The goals of this process are to decrease viscosity and the boiling point, elimination of metals, sulfur and other impurities and to increase the hydrogen‐to‐carbon (H/C) ratio. In general crude oil with high H/C and low heteroatomic content, requires less hydrogen for upgrading, resulting in a less expensive process. Metals and sulphurs have to be removed at some point during the upgrading and refining process because they may poison the chemical catalysts used and because the final fuel cannot contain these impurities. Heavy oil contains, in general, higher amounts of heteroatoms and organometallic constituents than crude oil; furthermore, the sulfur content of most crude oils is in the order of 1% by weight, whereas it can reach 5% in heavy oils (Speight and Ozum, 2001).

**Figure 1:**
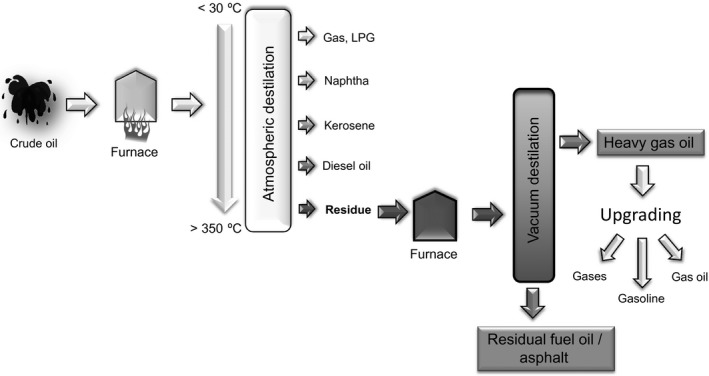
Schematic representation of initial process in a petroleum refinery.

The European Union has proposed an 80% cut in CO_2_ emissions by 2050 (https://ec.europa.eu/clima/policies/strategies/2050_en); this ambitious objective requires the transformation of the economy into more climate‐friendly and less energy‐consuming one. That means that new technologies have to be cleaner and more energy‐efficient. As in the EU, global trends advance towards a more sustainable economy. In this context, industrial biotechnologists have the responsibility to lead the way, transforming the old industries into cleaner and more efficient ones. This is precisely the type of contribution that Ismael and colleagues have reported in the paper published in this issue. They have taken a traditional industry (petroleum refinery) and have studied how they can transform one of the thermo‐chemical steps used to upgrade HVGO (energy intensive) into a biological process (carried out under environmentally friendly conditions) at the same time that they add value to the process by the production of biosurfactants (specifically rhamnolipids). Their approach is therefore ‘doubly green’; they intend to transform an energy intensive process into a less energy‐dependent one at the same time that they contribute to the reduction in the utilization of chemical surfactants by increasing the production of other more ‘environmentally friendly’ ones (rhamnolipids).

Interestingly, only one strain, *Pseudomonas putida* AK6U (Ismail *et al*., 2014, 2015), is responsible for the upgrading of HVGO and the production of rhamnolipids. The authors have demonstrated that this strain is able to grow using HVGO as carbon and sulfur sources at the same time that rhamnolipids (with the most abundant being rhamnosyl‐rhamnosyl‐β‐hydroxydodecanoyl‐β‐hydroxydodecanoate [Rha‐Rha‐C_12_‐C_12_] followed by rhamnosyl‐rhamnosyl‐β‐hydroxydecanoyl‐β‐hydroxydecanoate [Rha‐Rha‐C_10_‐C_10_]) are produced. For the successful industrial production of these compounds from HVGO not only growth conditions but also industrial purification of the compounds remained to be explored. It is known that rhamnolipid production is tightly regulated at different levels, with catabolite repression being one of the factors that affect its production; however in mixtures such as HVGO the presence of easily degraded carbon sources is not anticipated. High carbon‐to‐nitrogen ratios favour biosurfactant production and this ratio is also favourable in HVGO; the rhamnolipid yield obtained in this carbon and sulfur source is similar to that reported for other bacteria (9.8 g l^−1^ in 18 days).

The authors have also studied the differences in the HVGO composition during the growth of *P. putida* AK6U. HVGO is mainly composed of asphaltenes and maltenes. Asphaltenes represent the heavier compounds of crude oil and contain nitrogen, oxygen, sulfur, vanadium and nickel compounds as well as metal‐containing petroporphyrins and heterocycles; asphaltenes are formed of fused naphthenic and aromatic molecules with small aliphatic side‐chains and polar functional groups. Maltenes contain resins (small versions of asphaltenes), aromatic compounds and linear and branches hydrocarbons (Sharma *et al*., 2007; Speight, 2013 *). Pseudomonas putida* AK6U is able to slightly decrease the boiling point of the heavy fuel‐oil fraction with the concomitant increase in the proportion of lighter distillate diesel fraction. FT‐ICR‐MS analysis indicated that biological treatment increased the number of hydrocarbon and sulfur compounds in the maltene fraction of HVGO and decreased the amount of heavy fuel oil, suggesting the mobilization of asphaltenes to the maltene fraction. As the authors point out, the compositional differences between the biotreated and original HVGO are not sufficient to induce significant changes in the distillation profiles of the biotreated HVGO but ‘*on the industrial refining scale where huge volumes of the oil are processed, even small differences might lead to considerable increased yield, which further improves the revenue capture of the refining process’*. Interestingly, the authors have previously identified that *P. putida* AK6U is able to use compounds such as dibenzothiophene, benzothiophene, 3‐methyldibenzothiophene, 4,6‐dimethyldibenzothiophene and dibenzylsufide as sole sulfur sources (Ismail *et al*., 2014, 2015), results that point towards the elimination of organo‐sulfur compounds from the HVGO.

Obviously, there is still a long way to go to develop a complete and successful biotechnological process to substitute the thermo‐chemical technology, but this manuscript points out the possibility of upgrading HVGO by biological means. This is especially relevant nowadays, not only because it opens up a new clean technology for HVGO upgrading, but because this biotechnological process may, in principle, be applied to heavy oil. The term heavy oil commonly refers to oil that requires thermal stimulation to recover from the reservoir. Heavy oil is found all over the world and shares some of the properties of HVGO, such as being highly viscous, high molecular weight, low hydrogen‐to‐carbon ratio and with impurities such as nickel, vanadium, iron calcium, nitrogen, oxygen and sulfur. The International Energy Agency (IEA) estimated that approximately 6 trillion (6 × 10^12^) barrels of heavy oil are available worldwide, therefore, together with vacuum residue (VR) this oil is considered as an alternative source for fuel (Sahu *et al*., 2015). Despite the increase in the production of biofuels, oil demand is still expected to increase from 78 million barrels per day in 2002 to 118 million barrels per day in 2025; with the lighter products being the most demanded. New refineries will have to deal with upgrading residual fuel and with oil with decreasing quality (Hutzler *et al*., 2004); therefore petroleum refineries will be partially replaced by heavy or extra heavy oil refineries in the near future. Implementation of biological processes similar to those described by Ismail and colleagues will represent a unique opportunity for transforming the petroleum industry. However, there is still research ahead to achieve the final goal and roles have to be assigned to the game participants. There is no direct evidence about the role that biodegradation or/and biosurfactant properties play in the changes observed in HVGO after biological treatment; upgrading optimization will be more than desirable and studies about the incorporation of other strains to the process will probably follow this preliminary study. Other important aspects that should be further explored are those related with the rhamnolipid extraction in an industrial context and how to optimize yields. Technological implementation and administrative issues will have to follow. As an advantage for their exploitation, rhamnolipids and surfactins are the only commercially available biosurfactants, meaning that they have the approval of the regulatory authorities (Nitschke and Costa, 2007).

## Conflict of interest

None declared.
